# Hyperinflammation and Fibrosis in Severe COVID-19 Patients: Galectin-3, a Target Molecule to Consider

**DOI:** 10.3389/fimmu.2020.02069

**Published:** 2020-08-18

**Authors:** Juan Garcia-Revilla, Tomas Deierborg, Jose Luis Venero, Antonio Boza-Serrano

**Affiliations:** ^1^Departamento de Bioquímica y Biología Molecular, Facultad de Farmacia and Instituto de Biomedicina de Sevilla (IBiS), Hospital Universitario Virgen del Rocío/CSIC/Universidad de Sevilla, Seville, Spain; ^2^Department of Experimental Medical Science, Experimental Neuroinflammation Laboratory, BMC, Lund University, Lund, Sweden; ^3^Department of Experimental Medical Sciences, Experimental Dementia Research Laboratory, BMC, Lund University, Lund, Sweden

**Keywords:** COVID-19, cytokine storm, fibrosis, galectin-3, biomarker

## Abstract

COVID-19 disease have become so far the most important sanitary crisis in the XXI century. In light of the events, any clinical resource should be considered to alleviate this crisis. Severe COVID-19 cases present a so-called cytokine storm as the most life-threatening symptom accompanied by lung fibrosis. Galectin-3 has been widely described as regulator of both processes. Hereby, we present compelling evidences on the potential role of galectin-3 in COVID-19 in the regulation of the inflammatory response, fibrosis and infection progression. Moreover, we provide a strong rationale of the utility of measuring plasma galectin-3 as a prognosis biomarker for COVID-19 patients and propose that inhibition of galectin-3 represents a feasible and promising new therapeutical approach.

## Introduction

COVID-19 is a disease caused by the infection of a novel coronavirus known as SARS-CoV-2. COVID-19 has spread rapidly around the world causing a devastating pandemic with millions of people affected and thousands of lives gone (370,000 dead as per May 30, 2020) in what has become the main health and economic threat in the modern era. Most severe COVID-19 patients develop pneumonia and hyperinflammation likely related to a macrophage activation syndrome (1) commonly named “cytokine storm”. Linked to the inflammatory response, lung fibrosis emerges as a secondary event related to the progression of the pathology (2). Understanding the link between the hyperinflammation phase and fibrosis will give rise to new therapeutic targets especially beneficial in the most severe cases of COVID-19 (3). Given the pleiotropic roles of galectin-3 (gal3), especially those driving inflammatory-associated immune responses, fibrosis and hypoxia we propose the urgent need to decipher a potential pathological role of this lectin in severe cases of COVID-19 patients. Gal3 is a carbohydrate-binding protein expressed by macrophages, epithelial and alveolar cells in lungs (4). We would like to highlight three potential roles of gal3 in COVID-19 progression: (i) the macrophage-related hyperinflammation phase that drives the cytokine storm in most severe cases; (ii) the virus infection mechanism via the viral spike protein, given that its N-terminal domain has been suggested to evolve from a galectin origin (5); and (iii) the COVID-19-related lung fibrosis linked to the acute phase of diffuse alveolar damage, oedema, hypoxia, and inflammatory response. Therefore, gal3 encompasses unique COVID-19-associated pathophysiological features that deserve therapeutical attention associated with inflammatory response, infection mechanism, lung fibrosis and hypoxia. Importantly, the existence of clinically tolerable inhibitors for gal3 makes clinical trials feasible. Indeed, one of the gal3 inhibitors already available has proven its efficacy in Idiopathic lung fibrosis. Last, we wish to highlight the potential role of gal3 as a clinical biomarker tool. Measure of plasma gal3 levels has been used as a biomarker for several diseases with fibrotic or inflammatory features like heart failure. Here, we provide strong rationale to consider gal3 as a potential prognostic biomarker for severe COVID-19 cases.

## Galectin-3 May Play a Key Role in Pulmonary Associated Inflammatory Response and Lung Fibrosis

**FIGURE 1:**
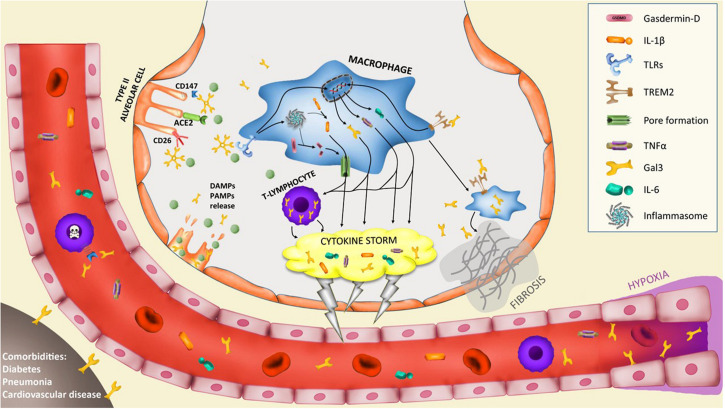
Potential implication of galectin-3 in the pathogenesis of severe COVID-19 cases. Infection of type II alveolar cells by SARS-CoV-2 is driven by ACE2-spike protein interaction and supported by CD147 and CD26 interaction. Gal3 can mediate ligand-receptor interaction by glycan recognition of spike protein and interaction with receptors. In particular, CD147-spike protein interaction promotes apoptosis of T-lymphocytes provoking systemic lymphocytopenia syndrome. Lysis of infected cells provoke the secretion of DAMPs and PAMPs recognized by alveolar macrophages receptors like triggering receptor expressed on myeloid cells 2 (TREM2) or toll-like receptors (TLRs) that trigger the upregulation of several cytokines including IL-6, TNFα and gal3. TLRs activation induces the assembly of the macromolecular platform known as inflammasome that promotes the cleavage of pro-IL-1β into active IL-1β and the cleavage of gasdermin-D (GSDMD), thus promoting pores formation in the cell membrane and induce the secretion of IL-1β. This macrophage activation triggers the hyperinflammation phase called “cytokine storm” and is supported by lymphocyte infiltration and proliferation with high expression of gal3 that contributes to the cytokine storm. The macrophage activation is composed of several phenotypes. A subset of macrophages develop a pro-fibrotic phenotype controlled by TREM2 and gal3 expression that triggers lung fibrosis. Moreover, hypoxia triggers gal3 expression by endothelial and smooth muscle cells. Lastly, comorbidities like diabetes, pneumonia or cardiovascular diseases increase basal levels of plasma gal3 what would contribute to COVID-19 severe prognosis.

SARS-CoV-2 primarily induces a lung inflammation during acute infection. The severity of the disease has been associated to lung infiltrating immune cells causing two well-defined features that are connected: (i) a lung hyperinflammation phase that becomes systemic through the progression of the pathology and (ii) the consequent lung fibrosis (6). In addition, the production of pro-inflammatory cytokines (cytokine storm) is believed to be a key event in COVID-19 mortality and morbidity. Hence, immune suppression should be carefully considered (7) due to the clinical evidence of deterioration found in some patients under this condition associated to the disease. Several publications have already pointed out that high levels of pro-inflammatory cytokines are in relation with the severity of the pathology (8, 9). Recently, single-cell RNAseq analysis performed on different immune cells in lungs from COVID-19 patients has provided valuable information about the immune response of the disease. In this study by Liao and colleagues (10), gal3 appears to be elevated in proliferative T cells associated to severe condition of COVID-19 patients. Moreover, a subset of macrophages express several markers associated to fibrotic processes like TREM2 or SPP1 (10), two markers that have been consistently associated to gal3 (11, 12). Indeed, a relevant study aimed at identifying the molecular mechanism involved in fibrosis identified a subset of pro-fibrogenic macrophages where gal3 was one of the most upregulated genes in association with TREM2 and SPP1 (13). Moreover, we have demonstrated a critical role of gal3 in microglial proinflammatory response and importantly, the ability of gal3 to further bind to and activate TREM2 (14) and TLR4 (15) both reported to be involved in lung disease and fibrosis. TREM2 is suggested to prevent macrophage apoptosis and promote chronic inflammatory disease after lung viral infection (16). TLR4 is a leading actor in the resolution of the inflammatory response in pneumonia (17) and TLRs are essential in the antiviral response triggering a strong inflammation involving interferon related genes, interleukins, chemokines as well as gal3 expression (18). So that, the mutually non-exclusive roles of gal3, TREM2 and TLRs in the COVID-19-associated hyperinflammation phase and the aforementioned lung fibrosis are plausible (see Figure 1). Supporting this, inhibition or genetic manipulation of TLR4 reduces the proliferation capacity of Influenza-A Virus and the pro-inflammatory response linked to pneumonia (19) and acute lung injury (20). Moreover, TLR4 has been associated to fibroblast activation and the subsequent lung fibrosis while fibroblast-specific deletion of TLR4 in mice induced substantial reduction in lung fibrosis (21). The latest is one of the main outcomes of the hyperinflammation phase associated with severe COVID-19 cases supported by the concomitant upregulation of gal3, TNFα and IL-6 in lobar and bronchial pneumonia (22). For instance, high levels of IL-1ra and IL-6 cytokines in plasma samples of COVID-19 patients demonstrate a clear association between the severity of the immune response and fatal outcome (9). Indeed, blocking IL-6 can be effective (23) and it has been suggested to ameliorate the severity of the pathology in some of the most critical cases (24). Moreover, previous SARS-CoV infection also runs with a strong inflammatory response and fibrosis (25–27) highlighting the importance of this approach for SARS-CoV-2. Mechanistically, it has been proved that SARS-CoV can activate NLRP3 inflammasome (28) something that occurs in H5N1 influenza infection as well in a gal3-dependent manner driving IL-1β production (29). Additionally, NLRP3 activation controls serum levels of gal3 (30). Notably, there are ongoing therapies aimed at slowing down the cytokine storm by infusing IL−1R antagonist (Anakinra) (31) and IL-6R antagonist (Tocilizumab) (31, 32). In our microglia work, we have demonstrated gal3 to govern both IL-1, IL-6 and TNFα release (14, 33). Gal3 thus emerges as a feasible pharmacological target to minimize the threatening hyperinflammation phase and subsequent lung fibrosis in COVID-19 patients.

## Galectin-3 as a Mediator for Viral Adhesion

Data obtained from SARS-CoV-2 genome revealed a spike glycoprotein as a key mediator of virus-host engagement and initiation of the infection (34). Indeed, novel glycation sites of this SARS-CoV-2 spike protein may explain the pandemic differences with previous SARS-CoV (35). Importantly, studies from N-terminal domain of spike protein determined that this domain likely evolved from a galectin precursor sharing most structural similarities with gal3 [see Ref. (36)] suggesting that gal3 could play a major role in SARS-CoV-2-host engagement. At present, only a possible relation has been suggested for coronavirus family and gal3. In an *in vitro* model of SARS-CoV-2 infection of human Caco-2 cells, gal3 protein levels increased 24 h after viral infection (37) what can be related to the increased transcriptional machinery due to its role in spliceosome, but also makes gal3 available for infections of nearby cells. The role of gal3 and glycation in virus infection has been observed in other viral infections like the influenza virus (38). Interestingly, gal3-virus interaction has been previously described in several viruses like herpesvirus-1 (HSV-1) infection, where gal3 binds to, at least, UL-46 viral protein promoting HSV-1 infection to host cells (39). Moreover, gal3-knockdown in keratinocytes decreased the infection of HSV-1, suggesting gal3 as a mediator of the viral infection process (39). Additionally, HSV-1 promotes the secretion of gal3 by the infected cells (40) exacerbating the initial infection. This mechanism also appears in diverse RNA-virus families. For instance, human immunodeficiency virus-1 (HIV-1) induces gal3 upregulation to later facilitate exosomes-mediated infection of nearby cells by its interaction with membrane fibronectin (41). Similarly, another retrovirus like HTLV-1 also takes advantage of gal3 to create a biofilm that facilitates the adhesion to new host cells (42). Moreover, influenza A virus and Streptococcus pneumonia bind to gal3 increasing their airway epithelial adhesion (43), suggesting a role of gal3 in primary and secondary airway infections in COVID-19 patients. In SARS-CoV-2 infection, angiotensin converting enzyme 2 (ACE2) has been proposed as the receptor for spike protein interaction (44). Despite no interaction has been described between gal3 and ACE2, gal3 has shown ability to bind to ACE (45) that shares the same extracellular domain with ACE2 (46), suggesting a potential interaction. Structural analysis of ACE2-Spike protein interaction has pointed out the relevance of ACE2 glycosylation (47). In addition, glycosylation of ACE2 has shown to be critical for viral invasion in previous coronavirus infections (48). Glycosylation sites also seem to be critical for the severity of SARS-CoV-2 infection in humans compared with other animal hosts (49). Moreover, a recent study identified at least two mutations in humans that incorporate new glycosylation sites in ACE2 receptor linked with an increased risk of COVID-19 (50). Nevertheless, other receptors have been proposed to interact with viral-spike protein like CD26 (34), a membrane glycoprotein previously involved in MERS epidemic (51), and CD147 (52), both known to bind gal3 (53, 54) (see Figure 1). Indeed, SARS-CoV-2 interaction with CD147 has been linked with apoptosis in T-lymphocytes what contribute to the leukocytopenia observed in severe COVID-19 patients (55) as part of the mechanisms of evasion from the immune system. In view of this data suggesting an involvement of gal3 in SARS-CoV-2 pathogenesis, clinical gal3 studies are urgently warranted to determine the clinical benefits of gal3 inhibition.

## Galectin-3 as a Biomarker and Therapeutic Target in COVID-19 Infection

As previously stated, lung fibrosis and hypoxia are a key feature related to the most severe cases of COVID-19 patients. Indeed, lung fibrosis has been found in autopsies in fatal COVID-19 cases (56). Lung fibrosis can be a secondary event consequence of the hyperinflammation phase in COVID-19. Both processes are connected mainly by the immune system and the activity of pro-inflammatory macrophage (1). Single-cell analysis has revealed a pro-fibrotic macrophage phenotype in the bronchoalveolar lavage fluid from patients with severe COVID-19 where lung fibrosis is present (10). Use of antifibrotic drugs has already been recently proposed [see Ref. (3)] as a treatment for severe COVID-19 patients. Among them, gal3 appears to be the most relevant due to its role in the inflammation and viral infection process. Gal3 antagonists have been developed against macrophage activation and inflammation associated to lung fibrosis showing positive results (57). Importantly, gal3 is already a therapeutic target in Idiopathic Pulmonary Fibrosis (IPF) with very promising results. Furthermore, a gal3 inhibitor (TD139, Galecto Biotech) has already passed phase I/IIa and proven its efficacy and safety (NCT03832946). We have successfully demonstrated the high efficiency of this drug in microglia cultures in response to well-defined pro-inflammatory agents (14, 15, 33). The availability of specific gal3 inhibitor in advanced clinical phases urge for clinical studies of the role of gal3 in SARS-CoV-2 pathogenesis. Hence, trying out interventions with gal3 inhibition could potentially be life saving for critically ill COVID-19 patients, and additionally support the lung tissue in the chronic phase for surviving patients.

Among the broad symptoms spectrum of COVID-19 patients, hypoxemia has shown the better correlation with disease severity. However, patients often maintain a “normal” lung function at the first stages of the disease while hypoxia appears as consequence of pulmonary vascular impairments that leads to a symptomatology that resembles the pulmonary arterial hypertension (PAH) (58, 59). Both COVID-19 and PAH develop hypoxia as one of their most relevant features and gal3 expression has been shown to be tightly regulated in hypoxic processes, making gal3 an attractive therapeutic target. For instance, Hao et al. (60) demonstrated the role of gal3 in hypoxia-induced PAH. Using small transferring RNA to inhibit gal3, they reduced the increased ventricular pressure, hypoxia-induced inflammatory response and alleviated ventricle hypertrophy of mice with hypoxia-induced PAH (60). Furthermore, Luo et al. corroborated that hypoxic condition in PAH triggers gal3 levels, promoting cell proliferation, inflammatory response and fibrotic driven tissue remodeling (61). In the same study, inhibition of gal3 reduced PAH (61). Similarly, Fulton and colleagues demonstrated that genetic or pharmacological inhibition of gal3 resulted in reduced pulmonary hypertension *in vivo* in a model of hypoxia-induced PAH (62). Recently, Fulton’s lab demonstrated that vascular smooth muscle cells are one of the main sources of gal3, which corroborates its implication in proliferation, apoptosis and fibrosis associated to PAH (63). Altogether, these studies highlight the importance of hypoxia-induced gal3 in PAH and lung function, being a potential target to counteract severe COVID-19 events.

At present, no clear prognosis biomarker have been identified while age and comorbidities are the unique clues in the prognosis of the disease. Considering that development of a hyperinflammatory phase is the main feature of the severe COVID-19 cases and given that immune cells can release gal3 during the inflammatory process (14, 15),we propose that it can be used as a biomarker of the inflammatory status in COVID-19 patients. Indeed, gal3 is elevated in plasma under strong inflammatory conditions (64). For instance, plasma levels of gal3 have shown strong correlation with disease progression in lung fibrosis (65) where post-mortem levels of lung gal3 can also correlate with the inflammatory status (66). Notably, cardiovascular diseases, diabetes or pneumonia are among the main risk factors for severe COVID-19 patients, all having in common elevated levels of gal3 (67, 68). Importantly, Mattioli and colleagues have shown that plasma from COVID-19 patients present elevated levels of gal3 (69) what supports that gal3 could be a good prognostic marker for severe COVID-19 and that elevated plasma levels of gal3 can participate in triggering the cytokine storm observed in severe COVID-19 patients. Consequently, plasma gal3 levels should be considered a clinical tool for inflammatory prognosis in COVID-19.

## Data Availability Statement

The original contributions presented in the study are included in the article/supplementary material, further inquiries can be directed to the corresponding authors.

## Author Contributions

JG-R and AB-S have contributed equally in the writing, design, and preparation of the manuscript. JV and TD have contributed in reviewing the manuscript. All authors contributed to the article and approved the submitted version.

## Conflict of Interest

The authors declare that the research was conducted in the absence of any commercial or financial relationships that could be construed as a potential conflict of interest.
